# Male fertility in *Pyricularia oryzae*: Microconidia are spermatia

**DOI:** 10.1111/1462-2920.16226

**Published:** 2022-10-20

**Authors:** Alexandre Lassagne, Sylvain Brun, Fabienne Malagnac, Henri Adreit, Joëlle Milazzo, Elisabeth Fournier, Didier Tharreau

**Affiliations:** ^1^ Plant Health Institute of Montpellier (PHIM), CIRAD Montpellier France; ^2^ Plant Health Institute of Montpellier (PHIM) University of Montpellier, CIRAD, INRAE, Institut Agro, IRD Montpellier France; ^3^ Institut Jacques Monod Université Paris Cité, CNRS Paris France; ^4^ Institute for Integrative Biology of the Cell (I2BC) Université Paris‐Saclay, CEA, CNRS Gif‐sur‐Yvette France

## Abstract

Sexual reproduction in Ascomycetes is well described in several model organisms such as *Neurospora crassa* or *Podospora anserina*. Deciphering the biological process of sexual reproduction (from the recognition between compatible partners to the formation of zygote) can be a major advantage to better control sexually reproducing pathogenic fungi. In *Pyricularia oryzae*, the fungal pathogen causing blast diseases on several Poaceae species, the biology of sexual reproduction remains poorly documented. Besides the well‐documented production of asexual macroconidia, the production of microconidia was seldom reported in *P. oryzae,* and their role as male gamete (i.e., spermatia) and in male fertility has never been explored. Here, we characterised the morphological features of microconidia and demonstrated that they are bona fide spermatia. Contrary to macroconidia, microconidia are not able to germinate and seem to be the only male gametes in *P. oryzae*. We show that fruiting body (perithecium) formation requires microconidia to get in contact with mycelium of strains of opposite mating type, to presumably fertilise the female gametes.

## INTRODUCTION

The reproductive system influences the apparition and the evolution of adaptive variants in response to selective pressures. Asexual reproduction induces clonality which allows an adapted genotype to spread quickly in a homogeneous environment. By contrast, sexual reproduction induces recombination which generates new genotypic combinations that can be advantageous in heterogeneous environments (De Meeûs et al., 2007; Stukenbrock & McDonald, 2008). Recombination also permits to slow down the accumulation of deleterious mutations (Bruggeman et al., 2003). Some eukaryote taxa like Ascomycete fungi exhibit a wide variety of reproductive systems, from strictly clonal to strictly sexual species, including numerous species that alternate both (Billiard et al., 2012). To understand how reproduction systems and genes involved in these processes evolved in Ascomycete fungi, a prerequisite is to document how sexual reproduction takes place and what the biological determinants of fertility are.

Sexual reproduction implies the succession of haploid, dikaryotic and diploid phases implemented during meiosis and syngamy (Billiard et al., 2012). In Ascomycete fungi, the reproduction cycle starts by the encounter of a male gamete (either contained in antheridia, or present as individualised specialised cells called spermatia) with a female differentiated gamete (the ascogonium), a step called fertilisation. The ascogonium is a multinucleate haploid cell produced by mitosis (Billiard et al., 2012) which differentiates specialised hyphae called trichogynes (Debuchy et al., 2010). Hormonal attraction directs the growth of trichogynes towards spermatia (Bistis, 1996). This hormonal chemotropism is controlled by the mating type genes on a pheromone/receptor recognition basis and occurs only between cells of opposite mating type in heterothallic species (Bistis, 1983). The contact between the trichogyne and the spermatia triggers cell fusion, that is, plasmogamy, after which the spermatia nucleus migrates across the trichogyne into the ascogonium (Brun et al., 2021). Plasmogamy is controlled by mating‐type genes (Bistis, 1996; Peraza‐Reyes & Malagnac, 2016). Plasmogamy is followed by syngamy, meiosis and differentiation of the ascus that will contain the ascospores produced after meiosis. Asci are contained in fruiting bodies (perithecia in *P. oryzae*), which are composed of an envelope of maternal origin that shelters the ascogonium. Hence, sexual reproduction in heterothallic Ascomycetes is an intricate process which requires, on the one hand, mating type compatibility between partners, and on the other hand, female and male fertile gametes.

*Pyricularia oryzae* (syn. *Magnaporthe oryzae*) is a phytopathogenic fungus of Pyriculariaceae (Klaubauf et al., 2014), causing blast disease on many cultivated cereals (rice, wheat, maize, millets…) and others Poaceae (Couch et al., 2005; Gladieux et al., 2018a). This fungus is responsible for the rice blast disease causing the loss of 3% of global rice yield (Savary et al., 2019). The genetic lineage of *P. oryzae* pathogenic on rice likely emerged in South Asia, close to the Himalayas (Gladieux et al., 2018b; Saleh et al., 2014; Tharreau et al., 2009; Zeigler, 1998) and is reported in more than 80 countries across the world (Boddy, 2016). The asexual mode of reproduction, through asexual spores (macroconidia) or mycelium multiplication, is commonly observed on rice host and most of the pathogen populations are clonal (Zeigler, 1998). Although sexual reproduction of *P. oryzae* has never been observed in the field on any host, evidences from biology, genetic and genomic studies of populations from rice suggest that sexual reproduction took place or is still taking place in limited areas in the putative centre of origin (Gladieux et al., 2018b; Saleh et al., 2012b; Thierry et al., 2021). *P. oryzae* is a heterothallic species with two idiomorphs at the mating type locus named Mat1.1 and Mat1.2 (Kanamori et al., 2007). Sexual reproduction is easy to complete in vitro in this species. Formation of perithecia is easily observed in the laboratory by crossing fertile strains of opposite mating types, on artificial medium (Nottéghem & Silué, 1992). However, only some strains are able to form perithecia, and are then defined as female fertile. Similarly, male fertility has so far been defined as the capacity to induce perithecia formation in a female fertile strain of opposite mating type (Saleh et al., 2012a). However, the mechanisms of fertilisation have not yet been described. By analogy with other Ascomycetes, microconidia have been supposed to be the male fertilising elements (Fukumori et al., 2004). Microconidia were observed for the first time by Kato et al. (1994). Microconidia as well as specialised hyphae bearing them (phialides) were also observed by Chuma et al. (2009) and more recently by Zhang et al. (2014). But the role of microconidia in fertilisation was not demonstrated yet. Here, we demonstrate that microconidia are fertilising cells, that is, spermatia in *P. oryzae*. We propose a new and direct biological definition of male fertility in *P. oryzae*, independent of the stimulation by a female fertile strain.

## EXPERIMENTAL PROCEDURES

### Biological materials

We used 12 *P. oryzae* strains isolated from six different host plants and collected in seven different countries (Table 1). Strains of both mating types (Mat1.1 and Mat1.2) were used to measure the production of microconidia. The strains CH0997 (Mat1.2) and CH0999 (Mat1.1) (Saleh et al., 2012a), used for the fertilisation and mating assays, were collected on *Oryza sativa* in China in 2008 and belong to lineage 1 (Gladieux et al., 2018b; Latorre et al., 2020; Thierry et al., 2021). All strains were stored at −20°C on dried filter paper (Valent et al., 1986).

**TABLE 1:** Production of microconidia by different *Pyricularia oryzae* strains in liquid culture. Information on synonymous names, on the sampling, on accession numbers to publicly available genetic information, on the fertility in mating assays and on the number of microconidia produced

Strain	Synonymous	References	Genetic information	Mating type	Isolated in	Isolated from	Lineage (rice strains only)	Mating assays		Microconidia production.ml^−1^		
								Male fertility	Female fertility	Replicate 1	Replicate 2	Mean replicates
US0068	70‐15	Dean et al. (2005)	INSDC Assembly GCA_000002495.2	Mat1.1	USA	Hybrid lab strain (pathogenic to rice)	1	Fertile	Fertile	2.36E+06	1.53E+06	1.94E+06
OG0002	UG771511, KA3	Kato et al. (2000)		Mat1.1	Ouganda	*Eleusine coracana*		Fertile	Fertile	8.57E+07	2.05E+08	1.46E+08
OG0003	UG771711, KA4	Tanaka et al. (2009)		Mat1.1	Ouganda	*Eleusine coracana*		Fertile	Fertile	7.03E+08	1.51E+09	1.10E+09
GY0006		This article		Mat1.2	French Guyana	*Oryza sativa*		Fertile	Fertile	2.80E+04	1.73E+04	2.27E+04
GY0011	GUY11	Leung et al. (1988)	NCBI database: ASM236848v1 (GY11 PacBio Bao 2016)	Mat1.2	French Guyana	*Oryza sativa*	1	Fertile	Fertile	1.04E+06	1.06E+06	1.05E+06
CD0141		This article		Mat1.2	Ivory Coast	*Leersia hexandre*		Fertile	ND	2.00E+04	2.13E+04	2.07E+04
TH0012		Gladieux et al. (2018a)	ENA repository: PRJEB8341 (TH16 GEMO)	Mat1.1	Thailand	*Hordeum vulgare*	1	Fertile	Fertile	6.13E+04	2.69E+06	1.38E+06
TH0016		Gladieux et al. (2018a)	ENA repository: PRJEB8341 (TH16 GEMO)	Mat1.2	Thailand	*Hordeum vulgare*	1	Fertile	Fertile	3.60E+04	4.40E+04	4.00E+04
CH0997		Saleh et al. (2012a, 2012b)		Mat1.2	China	*Oryza sativa*	1	Fertile	Fertile	5.33E+04	4.80E+04	5.07E+04
CH0999		Saleh et al. (2012a, 2012b)		Mat1.1	China	*Oryza sativa*	1	Fertile	Fertile	4.52E+05	5.49E+05	5.00E+05
BF0026		This article		Mat1.2	Burkina Faso	*Eleusine indica*		Fertile	Fertile	0.00E+00	0.00E+00	0.00E+00
CH0052		Gladieux et al. (2018a)		Mat1.1	China	*Oryza sativa*	2	Fertile	Sterile	0.00E+00	0.00E+00	0.00E+00

### Growing media

Rice flour agar (RFA) medium was prepared with 20 g of organic rice flour, 15 g of Bacto agar, 2 g of Yeast Extract (Bacto™) in 1 L water with 500,000 units of penicillin G added after autoclaving for 20 min at 120°C. Fresh homemade potato dextrose broth (PDB) was prepared with 200 g of sliced organic potatoes boiled for 30 min in 800 ml sterile water. The preparation was filtrated through multilayer gauze. The filtrated solution was added with 20 g of glucose and replenished to 1 L.

### Microconidia production

Microconidia were produced following a protocol modified by Zhang et al. (2014). Two circular plugs of 5 mm diameter of actively growing mycelium on RFA medium were used to inoculate 40 ml of fresh homemade PDB. The liquid culture was incubated for 3 days at 25°C, then 6 days at 20°C with permanent shaking at 150 rpm, and then filtrated on Miracloth (EMD Millipore Corp., 475855‐1R) to remove mycelium fragments, and centrifuged at 4500*g* for 10 min. The pelleted microconidia were resuspended in 1 ml sterile distilled water. When microconidia production was too low, after filtration on Miracloth, the liquid culture was filtrated at 0.45 μm and microconidia were collected from the filter surface by washing the filter in 3 ml sterile water. To evaluate the capacity of each studied strain to produce microconidia, two independent replicates were performed and microconidia were counted three times for each replicate using a Malassez cell of 5 mm^2^ and a limit threshold of 4000 units per ml.

### Staining and microscopic observation

For staining of cell wall, a droplet of Uvitex 2B (biovalley, 19517‐10) fluorescent dye was added directly in the suspension of macroconidia or microconidia on a microscopic slide and incubated in the dark for 15 min. The preparation was observed under photonic microscope Nikon ECLIPSE Ni‐E with 390 nm GFP excitation filter. Ascogonia were observed with an inverted microscope Zeiss Axio observer Z.1 (objective 63× Plan Apo ON 1.4 Oil DIC) and photographed with an ORCA Flash4 LT CMOS camera (Hamamatsu). Images were analysed using Fiji software (Schindelin et al., 2012).

### Mating assays

**FIGURE 1:**
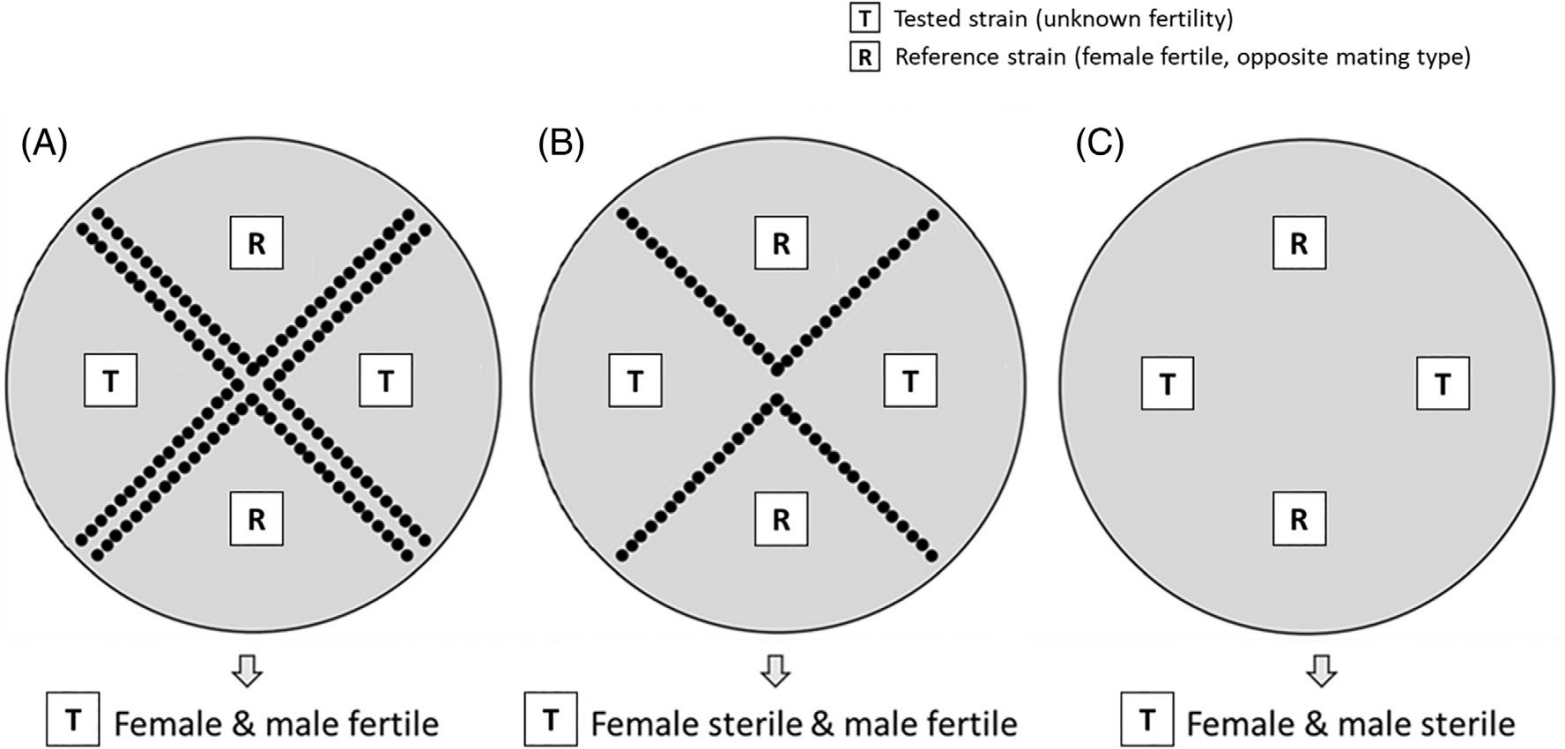
Interpretation of in vitro sexual crossing of *Pyricularia oryzae* with a tested strain of unknown fertility and a female fertile reference strain of opposite mating types. (A) Two lines of perithecia are formed: The tested strain is both male and female fertile. (B) One line of perithecia is formed: The tested strain is female sterile but male fertile. (C) Absence of perithecia: The tested strain is both male and female sterile.

Crosses between strains of opposite mating type were performed as described by Nottéghem and Silué (1992) on RFA medium in 90 mm Petri dishes. Plugs of mycelia grown on RFA at 25°C for 1 week were deposited on RFA medium following the design described in Saleh et al. (2012a). Cultures were incubated under continuous light for 2 days at 25°C and then at 20°C. The reference strains CH0997 and CH0999 are known to be female fertile when crossed with a broad number of genetically diverse strains (Thierry et al., 2021). The production of mature perithecia was evaluated 21 days after inoculation to determine the fertility phenotype according to Saleh et al. (2012b): the formation of two rows of perithecia between a tested strain and a reference strain of opposite mating type indicated that both strains were male fertile and female fertile (Figure 1A); the observation of a single row indicated that only the reference strain produced perithecia, and that the tested strain was female sterile but male fertile (Figure 1B); the absence of perithecia indicated that the tested strain was both female‐sterile and male‐sterile (Figure 1C). The cross CH0997 × CH0999 was used as positive controls, the crosses CH0997 × CH0997 and CH0999 × CH0999 were used as negative controls.

### Fertilisation assays

To assess the fertilisation capacity of microconidia, we first grew female fertile strains on RFA medium in 90 mm diameter Petri dishes for 3 days at 25°C and 6 days at 20°C. The two reference strains CH0997 (Mat1.2) and CH0999 (Mat1.1) were chosen as ‘female’ thalli in this experiment. After screening different strains, OG0002 (Mat1.1) was chosen as ‘male’ thallus in this experiment for its capacity to produce high quantities of microconidia. We prepared microconidia suspensions of OG0002 following the protocol described above, and adjusted them to 11 different concentrations: 400, 800, 1600, 3200, 4000, 6000, 8000, 12,000 and 24,000 microconidia.ml^−1^. For each suspension, 250 μl were sprayed on the mycelium of CH0997 and CH0999 strains. The inoculated plates were then cultured for 3 more days at 20°C before observation of perithecia formation. Two replicates were performed for each combination of female strain × concentration of microconidia suspensions.

The fertilisation capacity of macroconidia was tested in a similar way, using the same strains grown as described above. Macroconidia of strain OG0002 were harvested on aerial mycelium from cultures grown on RFA medium for 2 weeks at 25°C. To release macroconidia from the mycelium, cultures were gently scrapped after addition of 4 ml distilled water. Macroconidia suspensions were then filtrated on a 40 μm filter to eliminate mycelium fragments. Female fertile strains CH0997 and CH0999 were inoculated with 250 μl of suspensions at 9.00 × 10^4^ macroconidia.mL^−1^ of OG0002.

We also tested the fertilisation capacity of mycelium using the same strains as above (i.e., OG0002 as ‘male’, and CH0997 and CH0999 as ‘female’). To produce mycelium fragments of OG0002, 40 ml of commercial PDB (Difco™) were inoculated with two plugs of 5 mm diameter of mycelium actively growing on RFA medium. We observed that, in our conditions, microconidia are not produced in commercial PDB. The liquid culture was incubated with a permanent shaking at 150 rpm for 3 days at 25°C and then for 6 days at 20°C. The mycelium was then centrifuged at 4500*g* for 10 min and resuspended in 4 ml sterile water. Mycelium was sonicated for 1 min at 12 W and 20 Hz to obtain fragments. The female fertile strains CH0997 and CH0999, grown as described above, were then inoculated with 250 μl of mycelium fragment suspensions. The same volume of suspensions of mycelium fragments was also inoculated on two plates of RFA medium to check their viability.

### Quantification of perithecia on fertilised cultures

After 3 days of incubation, pictures of plates of fertilisation assays were taken with a camera. The proportion of the surface occupied by perithecia formed on plates was evaluated by image analysis with the software IPSDK Explorer developed by Reactiv'IP. This software uses machine learning techniques to classify pixels. The software was trained to classify pixels into three classes: background, mycelium and perithecia (model in Supporting Information). Due to the difficulty to precisely count individual perithecia, we quantified the number of pixels assigned to the ‘perithecia’ class. We then calculated the proportion of surface occupied by perithecia as the ratio of the number of pixels assigned to the ‘perithecia’ class on the number of pixels assigned to the ‘mycelium’ + ‘perithecia’ classes. We used the ‘drc’ R package (Ritz et al., 2015) to fit a log‐logistic model with the lowest value fixed to 0 and three parameters (*b*, *d*, *e*) as in the following equation:

$$f\left(x\right)=\frac{d}{\left[1+\exp\left(b*\left(\log(x\right)-\log\left(e\right)\right))\right]}$$

### Time frame assay for production of fertilisation competent female gametes

To determine the minimum time required for the female thallus to become competent for fertilisation, 20 μl of a suspension of OG0002 microconidia of opposite mating type (1 × 10^6^/ml) were poured on mycelial cultures of the female strain CH0997 of eight different ages (from 3 to 10 days). Eight plates of CH0997 were cultured on RFA medium at 25°C for 2–9 days, respectively, before being placed at 20°C 1 day before the inoculation with microconidia of OG0002. Perithecia formation was assessed every day from the 10th to the 17th day of the experiment. The presence of perithecia was assessed based on the observation of fungal structures with the morphological characteristics of perithecia.

## RESULTS

### Production of microconidia by *P. oryzae* strains

**FIGURE 2:**
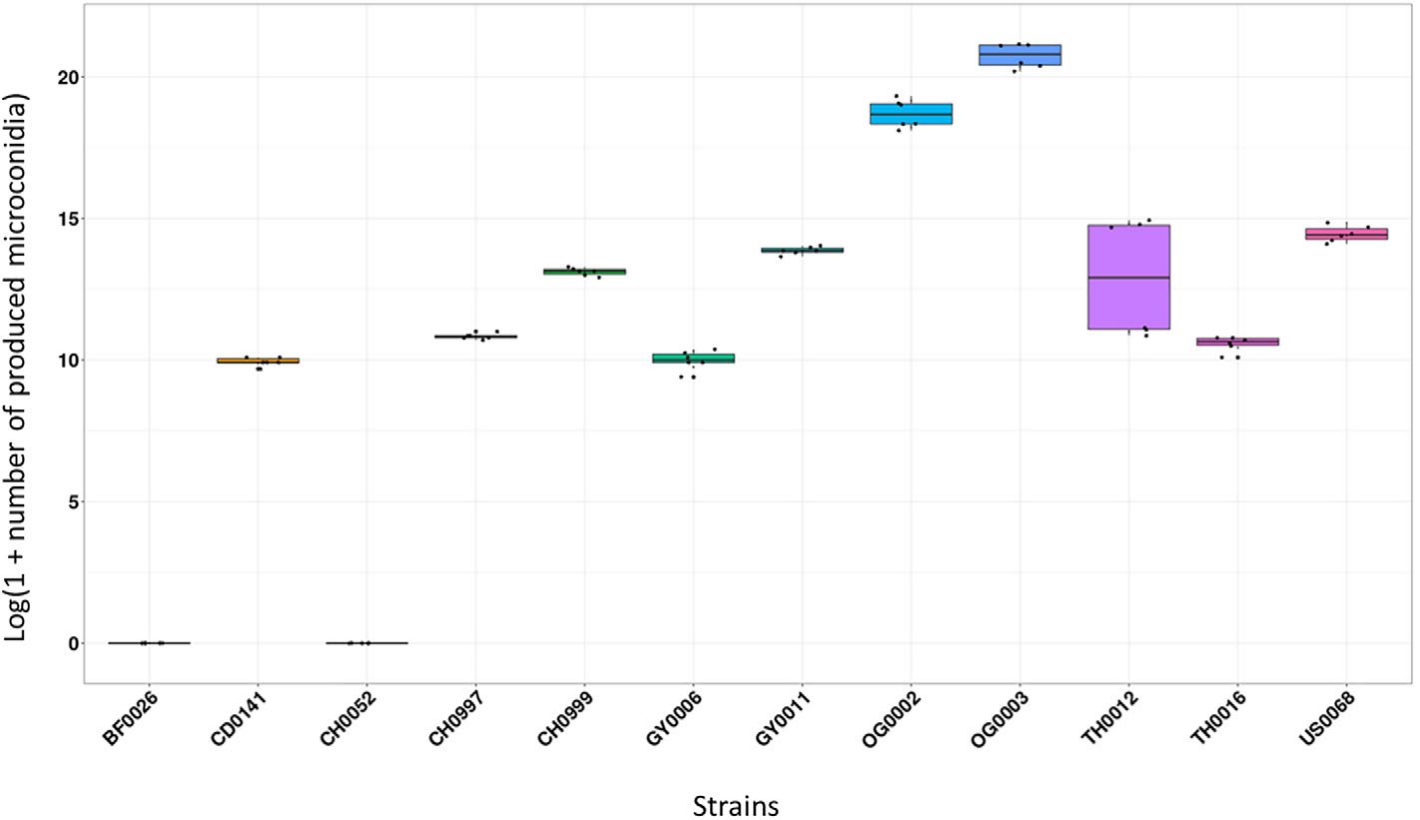
Quantity of microconidia produced for different strains of *Pyricularia oryzae* from different countries and host plants.

**FIGURE 3:**
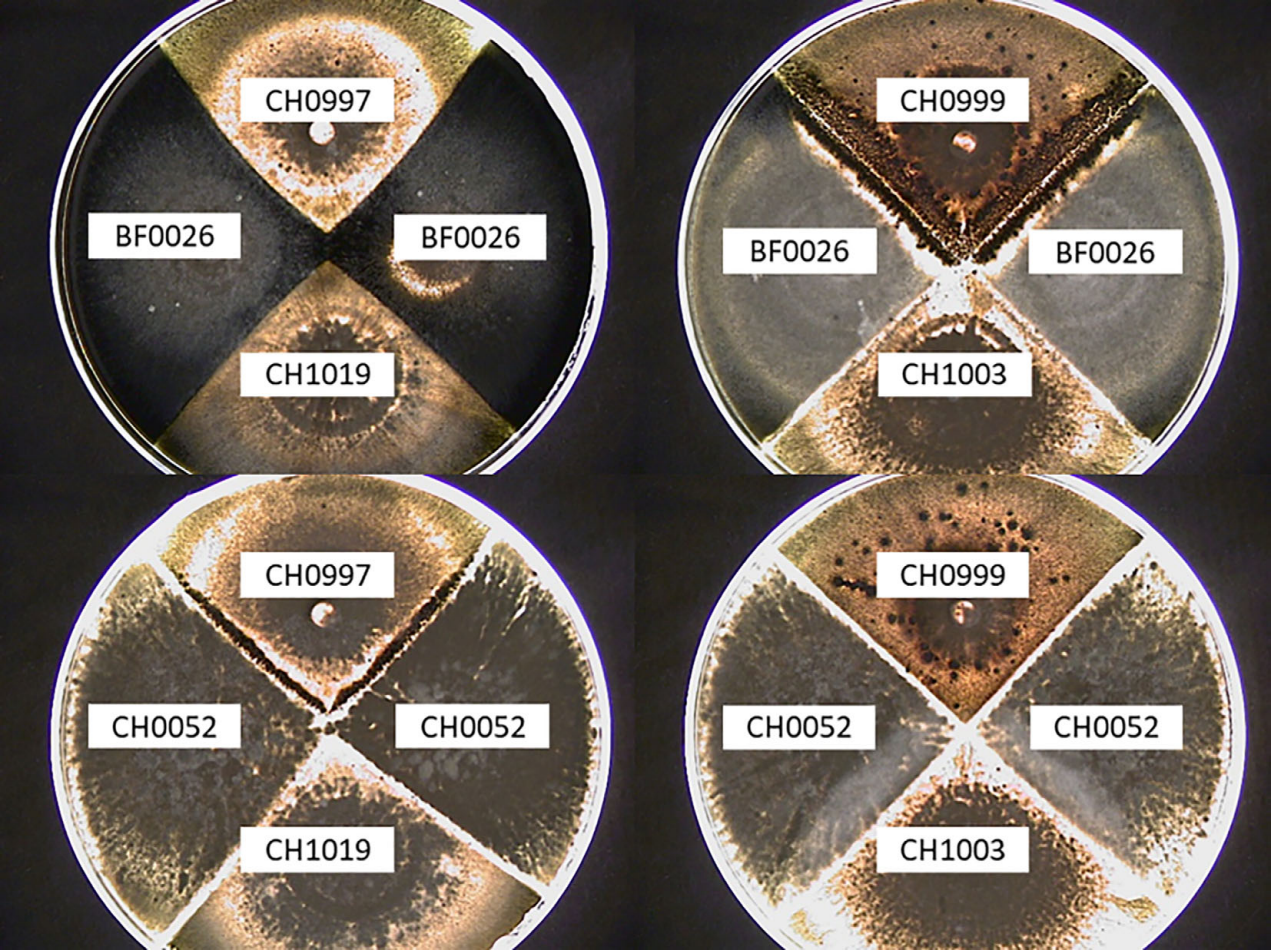
Crosses between strains CH0052 (Mat1.1) and BF0026 (Mat1.2) with four reference female fertile strains CH0999 (Mat1.1), CH1003 (Mat1.1), CH0997 (Mat1.2) and CH1019 (Mat1.2).

Ten out of the 12 studied strains produced microconidia in fresh homemade PDB (Table 1). All 10 strains inducing the formation of perithecia in classical in vitro tests produced from 2.07 × 10^4^ to 1.10 × 10^9^ (significant effect of the strain tested with a nested ANOVA: *F* = 1021.7, *p* = 2 × 10^−16^, *Df* = 11) microconidia (Figure 2). A significant difference in microconidia production was also observed when considering only the five strains isolated from the same host, rice (nested ANOVA: *F* = 232.57, *p* = 2 × 10^−16^, *Df* = 4). The two strains producing the highest number of microconidia were OG0002 and OG0003, with 1.46 × 10^8^ and 1.10 × 10^9^ microconidia.ml^−1^, respectively, that is, 100 to 10,000 times more than the other producing strains. Based on its high microconidia production, OG0002 was chosen as ‘male’ fertilising strain in the following assays. The number of microconidia produced was consistent between the two biological replicates of the same strain, except for TH0012. Although microconidia were not observed for strains CH0052 and BF0026 (Table 1), these strains induced the formation of a low quantity of perithecia on female reference strain. Furthermore, CH0052 and BF0026 induced perithecia formation with only one of the two female reference strains (Figure 3). As expected from previous reports, microconidia were produced regardless of the mating type of the strains, with four strains of each mating type producing microconidia and one strain of each mating type not producing microconidia. It is important to note that microconidia were obtained from cultures of single strains, confirming that the stimulation by another strain is not necessary to induce their production.

### Morphological characteristics of microconidia

**FIGURE 4:**
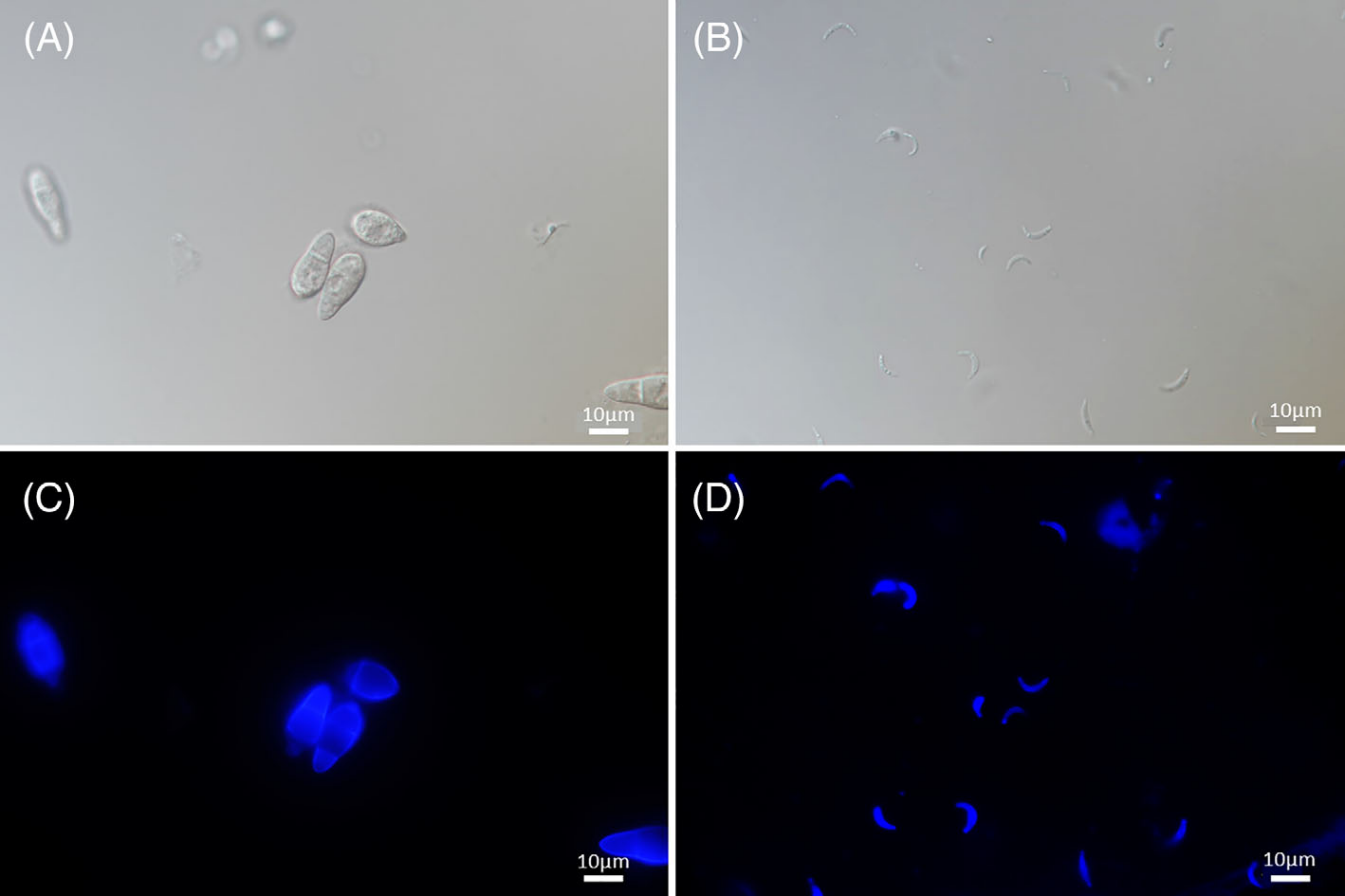
Macroconidia and microconidia of *Pyricularia oryzae* strain OG0002 under differential interference contrast light microscopy (100×) and with 0.05% Uvitex stain. (A) Macroconidia, (B) microconidia, (C) macroconidia (350 nm excitation light) and (D) microconidia (350 nm excitation light).

**FIGURE 5:**
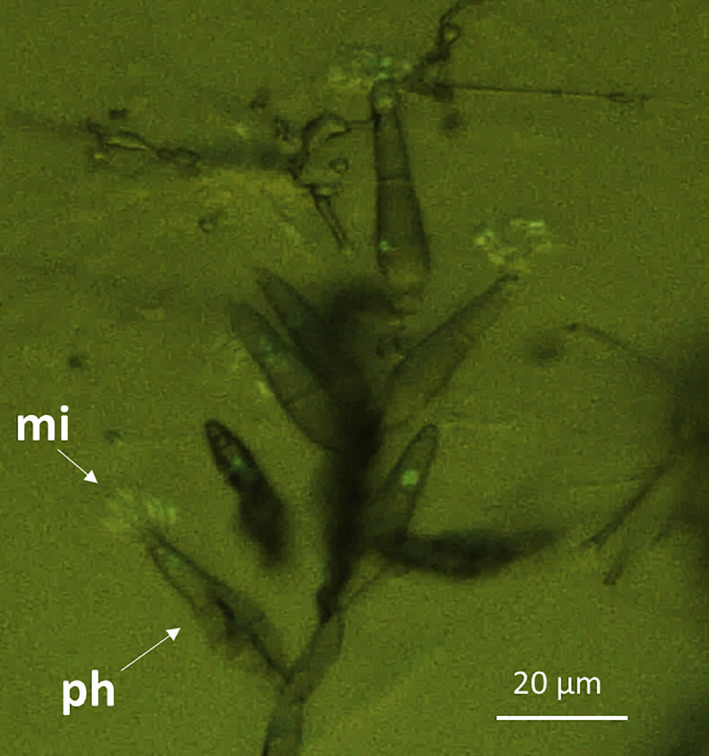
Fluorescence microscopy of microconidia worn by phialides hyphae of the transformed strain CH0997_GFP. (mi) microconidia, (ph) phialide.

The mean length of OG0002 microconidia was 7.36 μm (*n* = 62, standard deviation = 1.15). Hence microconidia are smaller than macroconidia whose dimensions are 16–33 μm long and 6–13 μm wide (Biju‐Duval, 1994; Chuma et al., 2009; Ou, 1985). Microconidia are also clearly distinguishable from macroconidia in their structure and shape (Figure 4): they are single‐celled and crescent‐shaped (Figure 4B,D), whereas macroconidia are three‐celled and pyriform with a characteristic light grey pigmentation (Figure 4A,C). Microconidia have a hyaline aspect (Figure 4B), which makes them uneasy to observe under microscope at low magnification and on cultures on solid medium. Both types of conidia have a chitinous cell wall evidenced by Uvitex staining (Figure 4C,D). Microconidia are worn by specialised hyphae called phialides (Figure 5).

### Fertilisation assays

**FIGURE 6:**
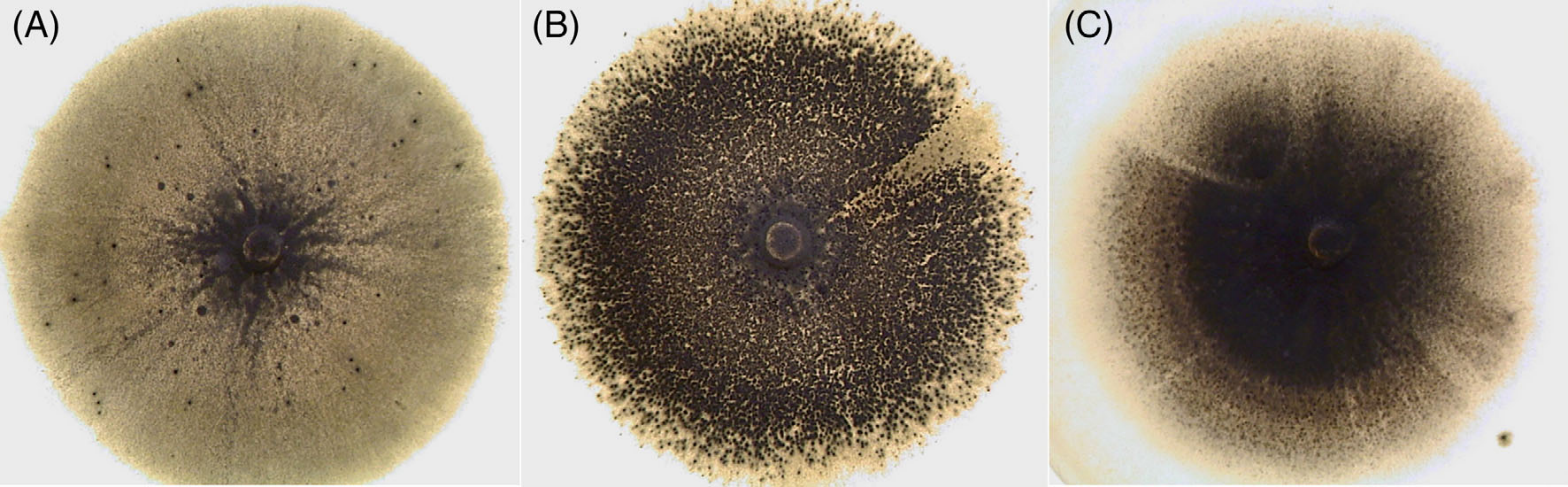
Observation of perithecia formation on female fertile strains 3 days after spraying with microconidia of OG0002 (Mat 1.1). (A) 100 microconidia sprayed on CH0997 (Mat 1.2) compatible strain. (B) 6000 microconidia sprayed on CH0997 (Mat 1.2) compatible strain. (C) 6000 microconidia sprayed on CH0999 (Mat 1.1) incompatible strain.

Spraying of pure suspension of microconidia of strain OG0002 (Mat1.1) on a ‘female’ strain of opposite mating type previously cultured for 9 days induced the formation of perithecia (Figure 6). Perithecia were formed on cultures of CH0997 (Mat1.2) for all concentrations of sprayed suspensions of microconidia in both replicates. Perithecia were observed 3 days after spraying. No perithecium was formed on cultures of CH0997 either sprayed with sterile water or unsprayed. As expected, no perithecia were formed by CH0999 (Mat1.1) after spraying with OG0002 microconidia, since both strains were of the same mating type.

**FIGURE 7:**
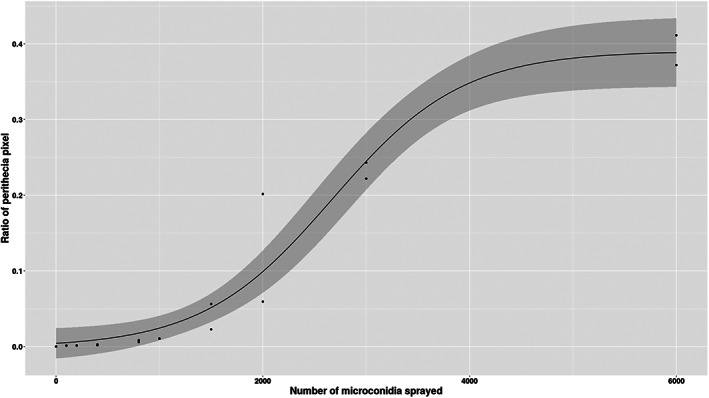
Relationship between the number of microconidia of OG0002 deposited and the number of perithecia formed on CH0997 is approximate by image analysis. The fitted model is a logistic curve with the lowest value fixed at 0 and 3 parameters (*b*, *d*, *e*).

We observed a significant positive relationship between the number of microconidia sprayed on the ‘female’ thalli and a proxy of the number of perithecia (i.e., the proportion of surface occupied by perithecia on the fertilised culture). The relationship between the number of microconidia sprayed and the proportion of surface occupied by perithecia was explained by a log‐logistic model with minimum value fixed at 0 and three parameters (*b*: slope, *d*: plateau, and *e*: ED50), with *b* = −1.59 × 10^−3^, *d* = 0.39 and *e* = 2670 (Figure 7). A plateau seems to be reached around 0.39 corresponding to 39% of pixels assigned to perithecia.

Unlike for microconidia spraying, no perithecia were formed 3 days after spraying with macroconidia or with mycelium fragments of OG0002 on CH0997. However, perithecia started to appear 2 weeks after spraying mycelium fragments or macroconidia. As expected, since they have the same mating type, no perithecia were formed at all after spraying with macroconidia or with mycelium fragments of OG0002 on CH0999.

### Production of fertilisation competent female gametes

**FIGURE 8:**
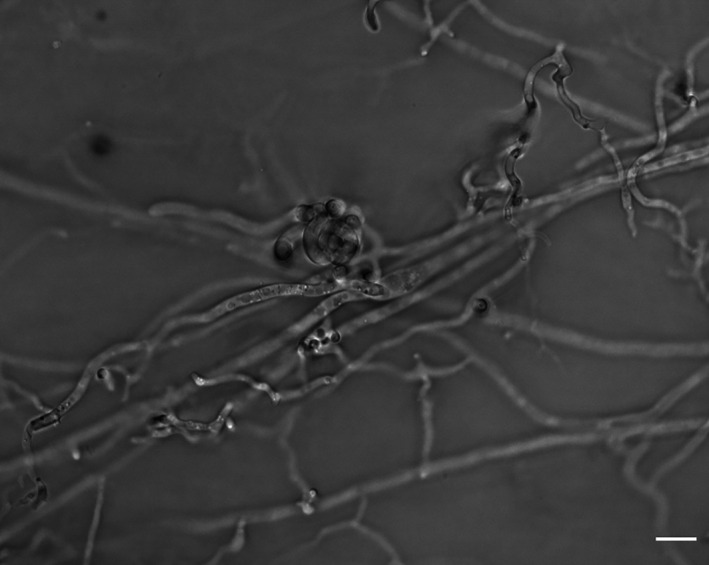
Inverted microscopy of ascogonium of the strain CH0997. Scale bar = 10 μm.

**FIGURE 9:**
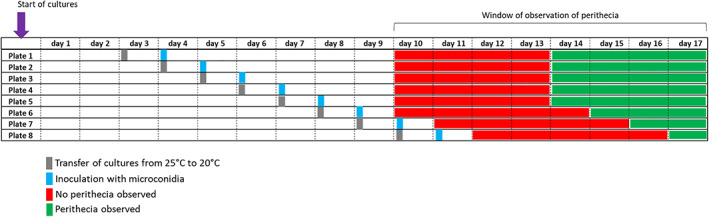
Time frame of perithecia development of strain CH0997 inoculated at different ages with spermatia of OG0002. Time scale corresponds to the number of days after the start of the culture of CH0997. The inoculation corresponds to the deposition of the microconidia suspension of OG0002 on the mycelial culture of CH0997.

Ascogonia were observed on female fertile strain thalli aged 11 days (Figure 8). To determine a putative minimum age for female receptivity, microconidia suspensions were sprayed on cultures of the female fertile strain CH0997 from 4 to 11 days after the starting of the culture. When the female fertile strain was grown for 7 days or more on RFA medium before inoculation (i.e., 6 days or more at 25°C and 1 day at 20°C), the formation of perithecia in those conditions occurred 6 days after spraying microconidia suspension. For younger cultures, that is, when the female fertile strain was grown 6 days or less on RFA medium before inoculation (i.e., 5 days or less at 25°C and 1 day at 20°C), it took more than 6 days for perithecia to be formed (7–10 days for cultures aged of 6–3 days, respectively; Figure 9). Hence, the age of the female thallus influences the ability to form perithecia.

## DISCUSSION

The first aim of our study was to confirm that *P. oryzae* from different origins were capable of producing microconidia. The other aim was to determine whether, in *P. oryzae* life cycle, microconidia could play the role of spermatia, that is, the biological elements inducing fertilisation and perithecia formation on compatible and competent strains.

Among the three previous studies describing microconidia, Chuma et al. (2009) and Zhang et al. (2014) focused on a single strain (G10‐1 and 70‐15, respectively), whereas Kato et al. (1994) tested 45 strains from different host plants, without quantifying microconidia production. In our experiments, 10 out of the 12 studied strains produced microconidia in quantities varying from 2.07 × 10^4^ to 1.10 × 10^9^ per ml. It is worth to note that strains from both mating types were capable of producing microconidia. Furthermore, the capacity of producing microconidia did not seem to depend on the original host of the strain either, since strains collected from *Hordeum vulgare*, *Eleusine coracana* or *Oryza sativa* produced microconidia. Morphological observations of *P. oryzae* microconidia confirmed previous studies (Chuma et al., 2009; Kato et al., 1994): they are crescent‐shaped hyaline and are two to four times smaller than macroconidia, which makes their observation harder and could explain why they were so scarcely reported in the literature.

Fertilising cells in Ascomycetes can be either unspecialised cells like macroconidia or mycelial fragments, or specialised cells, that is, microconidia (Pöggeler et al., 2018). Microconidia were proved to be spermatia in several Ascomycete species, including *Botrytis cinerea* (Fukumori et al., 2004) and *Neurospora crassa* (Maheshwari, 1999). In these two species, however, microconidia designate the male gametes, are able to germinate, and are distinct from macroconidia (often called conidia) which are asexual spores but can also be fertilising elements (Brun et al., 2021); hence both microconidia and macroconidia are spermatia in *B. cinerea* and *N. crassa*.

In *P. oryzae*, the role of microconidia as spermatia is commonly accepted, but this had never been demonstrated so far (Chuma et al., 2009; Zhang et al., 2014). Here, we demonstrate that in *P. oryzae*, microconidia are the only fertilising elements, hence that they are spermatia. Contrarily to macroconidia and mycelium fragments, only microconidia sprayed on a compatible female fertile strain led to the formation of perithecia after only 3 days. In addition, the number of perithecia formed was positively correlated to the number of microconidia sprayed, confirming that microconidia have the capacity to induce perithecia formation. Reaching a plateau of perithecium formation despite growing numbers of sprayed spermatia suggests that the number of female gametes competent for fertilisation could be the limiting parameter. Although no microconidia were observed for two strains (CH0052 and BF0056), they induced the formation of perithecia on female fertile strains of the opposite mating type. We hypothesised that the two strains produce quantities of microconidia that are below the detection threshold of the counting method, that is, 4000 microconidia per ml. The apparition of perithecia after spraying macroconidia or mycelium fragments of the male strains on a female‐compatible strain was clearly delayed (15 days compared to 3 days after spraying microconidia). We hypothesised that the female strain has been fertilised by microconidia produced de novo by new thalli resulting from the growth of mycelium fragments or macroconidia germination. However, we cannot exclude that a yet unknown alternative way of fertilisation may take place when macroconidia or mycelium fragments are sprayed.

We observed that the age of the female thallus influenced its ability to produce perithecia when inoculated with spermatia of a compatible strain (Figure 9). In our conditions, a minimum age of 7 days of culture is required for female thalli to be able to produce perithecia when sprayed with spermatia. This minimum age could correspond to the time needed for female gametes to differentiate. In *Podospora anserina*, male and female gametes start to differentiate after 3 days of growth on minimal medium at optimal temperature (Coppin et al., 1997; Silar, 2011). In *P. oryzae*, other parameters such as temperature or nutrient supplies might probably also play a role on the time needed for female thalli to become competent.

Before the present study, microconidia had never been observed in our laboratory conditions despite successful in vitro crossing between strains. One explanation could be the low quantity of microconidia produced on RFA medium (the medium routinely used for growing strains and performing in vitro crosses; Nottéghem & Silué, 1992). Here, we used the modified protocol of Zhang et al. (2014) to obtain high quantity of microconidia. Sporulation is a highly variable developmental process subjected to many environmental parameters. In *N. crassa*, several external factors such as humidity, temperature or starvation have an effect on male gamete production (Debuchy et al., 2010; Maheshwari, 1999). For instance, a low concentration of carbon source and a shift from high to low temperature also facilitates the production of microconidia in *N. crassa* (Ebbole & Sachs, 1990; Rossier et al., 1977). The production of microconidia in *P. oyyzae* might also rely on specific conditions, which might be related to the fact that sexual reproduction was seldom observed in nature.

Ten strains that produced microconidia in our conditions showed significant differences in the quantity of microconidia produced. The strains OG0002 and OG0003, that were isolated from *Eleusine corocana* in the same field, produced more microconidia than other. Confirming the difference of production of microconidia between strains from different host‐specific groups will require additional studies. However, differences within host‐specific groups of *P. oryzae* are also worth documenting. For example, in this study, we observed significant quantitative differences in the production of microconidia between strains isolated from rice. Male fertility likely depends not only on the original host but also on the reproduction mode of the populations or genetic groups sampled. If spermatia have no other role than fertilisation, their production is expected to decrease and to be lost in populations that are no longer experiencing sexual reproduction. *P. oryzae* populations from rice allow to test this hypothesis and to investigate the heritability and genetic bases of microconidia production.

The demonstration of the role of microconidia as spermatia allows to reconsider the definition of male fertility in *P. oryzae*. Until now, male fertility was only defined as the capacity of a strain to induce perithecia formation on a strain of opposite mating type in a cross (Nottéghem & Silué, 1992; Saleh et al., 2012a). There are several limitations to this initial definition. The use of a female auxiliary strain is necessary to assess male fertility, which introduces an additional variable parameter. A second limitation is the potential existence of pre‐zygotic barriers. Here, we define male fertility of *P. oryzae* strains as the ability of a strain to produce male gametes (i.e., microconidia), and without crossing with a reference strain. Counting the spermatia produced will in addition permit a quantitative phenotyping of male fertility.
